# The Role of Vaccination in Adult Solid Organ Transplantation: Updated Reviews with Recent Guidelines

**DOI:** 10.3390/microorganisms14010194

**Published:** 2026-01-15

**Authors:** Girish Mour, Sujay Dutta Paudel, Pranav Modi, Umesh Goswami, Jamilah Shubeilat, Lucy Ptak, Sandesh Parajuli

**Affiliations:** 1 Department of Medicine, Mayo Clinic, Phoenix, AZ 85054, USA; mour.girish@mayo.edu (G.M.);; 2 Department of Medicine, Feinberg School of Medicine, Northwestern University, Chicago, IL 60208, USA; sujay.paudel@nm.org; 3 Internal Medicine, North Mississippi Medical Center, Tupelo, MS 38801, USA; 4 Kansas College of Osteopathic Medicine, Kansas Health Science University, Wichita, KS 67202, USA; 5 Division of Nephrology, Department of Medicine, University of Wisconsin School of Medicine and Public Health, Madison, WI 53705, USA

**Keywords:** vaccine, solid organ transplant, post-exposure prophylaxis, COVID-19 vaccination, travel-related vaccination

## Abstract

Vaccination remains a cornerstone of infection prevention in adult solid organ transplant (SOT) recipients, a population at heightened risk for vaccine-preventable diseases due to chronic immunosuppression and comorbidities. Updated guidelines from the American Society of Transplantation Infectious Diseases Community of Practice (AST IDCOP) and other international bodies emphasize the need for timely and comprehensive vaccination strategies before and after transplantation. This review synthesizes current literature and practice guidelines on vaccination in adult solid organ transplant (SOT) candidates and recipients. Published peer-reviewed studies, clinical trials, and consensus guidelines were evaluated, with emphasis on vaccination timing, safety, immunogenicity, dosing strategies, and serologic response monitoring in the SOT population. Comprehensive vaccination planning before transplantation, combined with appropriate post-transplant booster strategies, remains vital to improving long-term outcomes in SOT recipients. This review provides clinicians with an updated, evidence-based framework for integrating evolving vaccination guidelines into the care of adult transplant patients.

## 1. Introduction

Immunization can avert illnesses that carry a significant risk of death and their associated complications in individuals with compromised immune systems [1]. Adult solid organ transplant (SOT) recipients are at markedly increased risk for vaccine-preventable infections due to chronic immunosuppression and comorbid conditions [2,3]. The American Society of Transplantation’s Infectious Diseases Community of Practice (AST IDCOP) recommends reviewing and updating immunizations pre-transplant, with inactivated vaccines administered at least two weeks prior to transplantation and live-attenuated vaccines at least four weeks before transplantation [4,5].

The current review provides details of comprehensive care for transplant candidates, both pre- and post-transplantation, discussing specific scenarios like the elderly population.

## 2. Pre-Transplant Vaccination and Timing

Patients with end-organ dysfunction/failure are at significantly increased risk of infections due to reduced innate and adaptive immune responses [6]. Vaccination provides a significant intervention, as some of these could be life-saving. Concerns remain regarding the efficacy of vaccination due to reduced seroconversion rate, reduced duration of effective protection, timing of vaccination in relation to organ transplantation, and the need for different dosing strategies compared to the general healthy population. For example, as chronic kidney disease (CKD) progresses, the efficacy of hepatitis B vaccinations also decreases. In healthy adults, seroconversion rates exceed 95%. In comparison, the rates drop to about 90% in patients with CKD stages 3–4, and further decline to only 40–50% in those with end-stage renal disease (ESRD) [7].

As a general recommendation, proper full vaccination should be performed at least 4 weeks prior to organ transplantation for live vaccinations and at least 2 weeks before organ transplantation for inactivated vaccines [4,5].

### 2.1. Influenza

Influenza is associated with significant morbidity in SOT recipients. This includes higher rates of hospitalization and intensive care unit admission, increased mortality, and association with impaired allograft outcomes [8]. Annual vaccination with the seasonal influenza vaccine for all patients with chronic medical conditions, including patients on dialysis, is highly recommended [9,10]. Currently, influenza vaccines come in an inactivated injectable form and the live attenuated form. Specifically, the high-dose regimen is approved for individuals 65 years and older; otherwise, there is no specific preference for any vaccine for adults [9]. Vaccination is recommended to be started in September or October, though it can be given throughout the influenza season. Early vaccination is not recommended due to the possibility of waning response. Booster dosing is also not recommended.

### 2.2. Pneumococcal Vaccine

As per the United States Advisory Committee on Immunization Practices (ACIP), all solid organ transplant candidates and recipients should be vaccinated against Streptococcus pneumoniae [11]. There are four types of pneumococcal vaccines: three are conjugate vaccines (PCV15, PCV20, PCV21) and one is a polysaccharide vaccine (PPSV23). The number denotes protection against types of pneumococcal bacteria.

On 17 June 2024, the Food and Drug Administration approved the 21-valent pneumococcal conjugate vaccine PCV21 for adults aged ≥19 years. While current guidelines do not specifically address the administration of PCV21 in immunocompromised individuals, we propose that PCV21 may be considered a viable option for this patient population.

For immunocompromised patients, including patients with SOT, the ACIP recommends that patients aged >19 years receive a single dose of the PCV21 vaccine. If PCV21 is not available, PCV20 alone or PCV15 followed by PPSV23 ≥ 1 year later are recommended alternatives. Table 1 provides details regarding the vaccination schedule in different age groups and based on vaccination status.

### 2.3. Hepatitis B

Patients with end-stage organ dysfunction, especially the ESRD population, are already at an increased risk of hepatitis B infection due to contact with healthcare workers, equipment contamination, dialysis patient population, and the need for blood transfusions [12]. Hepatitis B vaccination (HBV) in end-stage liver disease yields seroconversion rates of between 44 and 54%, compared to over 90% in healthy controls—a decline attributed to both disease severity and immunosuppressive therapy [13]. One recent study showed that patients with chronic liver disease who were vaccinated against hepatitis B had a low seroconversion rate of 35% [14].

Currently, hepatitis B vaccination in the United States is yeast-derived: the conventional hepatitis B vaccine, Recombivax HB and Engerix-B; and the recently approved Heplisav-B, derived using recombinant hepatitis B antigen with adjuvant cytidine–phosphate–guanosine (CpG). Conventional vaccines require a three-dose regimen for 6 months, while the Heplisav-B vaccine needs a two-dose regimen over 1 month apart [15].

Dosing strategies for the ESRD population, as compared to other organ dysfunction, are different. In this patient population, strategies such as doubling the vaccine dose or administering booster doses may be considered [16]. There are two recommended hepatitis B vaccination regimens for adults in ESRD patients on dialysis:

- Recombivax HB (dialysis formulation, 40 mcg): Administered as a series of three doses at 0, 1, and 6 months.
- Engerix-B (double dose, 2 mL = 40 mcg): Given as four separate doses at 0, 1, 2, and 6 months.

The Heplisav-B vaccine can be administered one month apart without any change in dosage. In a multicenter trial, CKD patients who received Heplisav-B maintained longer persistence of antibody levels ≥ 100 mIU/mL than those receiving Engerix-B. The geometric mean titers (GMTs) of anti-HBs were significantly higher in the Heplisav-B group (*p* ≤ 0.0001) [17]. Another study evaluated four doses of Heplisav-B [18]. A high seroprotection rate of 89% (anti-HBs ≥ 10 mIU/mL) was achieved by week 20, with 81% achieving antibody titers ≥ 100 mIU/mL. The geometric mean concentration of anti-HBs reached 1061 mIU/mL, indicating robust immunogenicity in this population. Heplisav-B was well tolerated, with no significant safety concerns reported. It is recommended to give two doses of Heplisav-B and review the response [18]. We suggest that if there is an inadequate response with two doses, then another two doses can be administered. Currently, Heplisav-B is not approved for patients on dialysis as the safety and effectiveness of Heplisav-B have not been established in this patient population. In subjects with liver disease, Heplisav-B was more effective in providing a better seroconversion (45% with Engerix-B as compared to 63% with Heplisav-B) [19]. Heplisav-B resulted in a 2.7 times increased likelihood of achieving seroconversion. As per AST IDCOP Guidelines 2019, patients undergoing liver or heart transplantation can also receive hepatitis B vaccination at an accelerated schedule (either at 0, 1, and 2 months or at 0, 7, and 21 days) [4,20].

It is important to check for the response rate to hepatitis B vaccination 1–2 months after finishing the recommended scheduled series. Lack of response may require another booster series [21,22,23]. If there is still no response to the booster series, then further attempts to vaccinate should not be made. It is prudent to check the antibody titer periodically pre-transplantation and after transplantation, as immunity can wane, requiring additional doses or readministration with the entire series [21,22,23].

### 2.4. Herpes Zoster

Compared to the general population, patients with CKD have a significantly higher risk of herpes zoster infections [24,25]. There are two vaccine formulations available: the live attenuated vaccine (zoster vaccine live [ZVL], which is no longer available in the United States) and the recombinant glycoprotein E vaccine (recombinant zoster vaccine [RZV], marketed as Shingrix). The Centers for Disease Control (CDC) recommends the recombinant zoster vaccine (RZV, Shingrix) for adults aged 19 years and older who are immunodeficient or immunosuppressed, which includes organ transplant candidates and recipients. Two doses of RZV are recommended, administered 2–6 months apart. For those who might benefit from a shorter interval, the second dose can be given 1–2 months after the first. A systematic review and meta-analysis found that the herpes zoster vaccine (HZV) is effective and has a low adverse event profile in patients with CKD. The effectiveness is strongest in non-dialysis CKD patients [26]. Similar recommendations are applied to patients with other organ dysfunctions.

### 2.5. Hepatitis A

Transmission of the hepatitis A virus is through the fecal–oral route, especially in countries with low sanitation standards. In a small study assessing the response of the hepatitis A vaccine in patients with ESRD, antibody response was almost 100% in those who received the vaccine intramuscularly and was well tolerated [27]. Similarly, hepatitis A infection in patients with chronic liver disease can carry significant morbidity and mortality due to underlying liver dysfunction. The response rate may depend on the stage of liver disease, with compensated liver disease having a higher seroconversion rate (71%) compared to decompensated liver disease (37%), and an increased response with the booster/second dose [28,29]. The recommendation is to follow up with the same dosing schedule for the general population.

### 2.6. Respiratory Syncytial Virus (RSV)

RSV is recommended for the patient population aged 60–74 who are at higher risk for developing severe disease and those older than 75 years old [30]. No booster dosing is required as a single dose is protective for at least two seasons. We do not have data regarding additional dosing of the RSV vaccine beyond a single dose. Limited data show that the vaccine may be effective in patients with underlying medical conditions, with an efficacy rate above 80% based on the underlying comorbid condition [31]. It is our opinion that the RSV vaccine should be considered in subjects who are candidates for transplantation, irrespective of age, as long as insurance coverage is available.

### 2.7. Tetanus–Diphtheria (Td) and Tetanus–Diphtheria–Acellular Pertussis (TdaP) Vaccines

The general recommendations for the Tetanus Toxoid, Reduced Diphtheria Toxoid, and Acellular Pertussis (TdaP) vaccine are similar to those for the general population.

Persons aged ≥19 years who have never received a dose of TdaP should receive one dose of TdaP, followed by a second dose four weeks later and a third dose 6–12 months after the second dose. To maintain ongoing protection against tetanus and diphtheria, one booster dose of either Td or TdaP should be given every decade throughout their lives [32].

### 2.8. Meningococcal Vaccination

Two types of meningococcal vaccination are available, targeting different serotypes: MenACWY is a quadrivalent vaccine (A, C, W, and Y), and MenB targets the B serotype. The vaccination strategy should be guided by prior vaccination status and ongoing risk.

For meningococcal vaccine-naïve adults at increased risk, a two-dose primary series of MenACWY administered 8 weeks apart is recommended, followed by a booster dose every 5 years if risk persists. MenB vaccination should be administered as a two- or three-dose series, depending on the product used.

For individuals previously vaccinated with MenACWY, a booster dose is recommended every 5 years while risk remains. MenB boosters may be considered every 2–3 years in patients, if the risk remains.

### 2.9. Live Vaccines

Rates of seronegativity to measles, mumps, rubella (MMR), and varicella remain high at around 32% pre-transplantation [33]. If MMR serology is negative, the vaccine should be administered at least 4 weeks prior to transplant. A repeat dose can be given if the patient remains seronegative. For individuals who have never received a varicella-containing vaccine, a two-dose series should be administered, with the doses spaced 4 to 8 weeks apart. If the individual has previously received only one dose of a varicella-containing vaccine, a second dose should be provided at least 4 weeks after the initial dose to ensure adequate immunization.

## 3. Post-Transplant Vaccination, Including Timing

Vaccination plays a vital role in preventing infections following SOT. Strategies are individualized, mainly influenced by the net degree of immunosuppression, organ type, age, recent infections, and rejection episodes. Data is lacking in adult solid organ transplantation regarding the need for monitoring the persistence of serological response post-transplantation in patients who have received pre-transplant vaccination. However, in general, patients are more likely to remain positive based on pre-transplant response and the timing of vaccination. Ideally, vaccination should be completed pre-transplant. The following section outlines the post-transplant vaccination schedule for patients who did not complete the recommended pre-transplant vaccinations; the schedule may differ for those who received their vaccines prior to transplantation [34,35]. It is generally agreed to avoid vaccinations in the immediate post-transplant period [36] as vaccine efficacy may be decreased due to impaired immune response with a high degree of immunosuppression [35]. The AST IDCOP recommends that inactivated vaccines can be administered starting at 3 months post-transplant [4]. We assume that there is variability across transplant centers regarding the timing of vaccination post-transplantation based on post-transplant events, type of vaccination, and possible concerns for potential vaccine-related complications [34,35].

Vaccination in SOT recipients typically includes a combination of inactivated vaccines and certain non-live vaccines, which are deemed safer compared to live-attenuated vaccines (Table 2).

### 3.1. Influenza

Influenza vaccination in SOT recipients is associated with reducing the morbidity and mortality associated with influenza infection [51]. Lung transplant recipients face unique challenges with higher rates of viral infection, like influenza infection, compared to other SOT groups, due to increased exposure to the environment. Lung transplant recipients are also at a higher risk of developing secondary complications, including chronic lung allograft dysfunction [52,53]. Timing is crucial, with recommendations to administer the vaccine at least one month post-transplant to achieve good outcomes without compromising graft function [4]. In a multicenter trial, receiving annual influenza vaccination was associated with a decreased incidence of pneumonia by 65% and requiring ICU admission by 51% [54]. The American Society of Transplantation (AST) recommends annual inactivated influenza vaccination for all SOT recipients and their close contacts [55]. Studies have shown no concern for increased risk for allograft rejection from influenza vaccination. Both the standard and high-dose influenza vaccines have been well tolerated in SOT recipients [56,57]. It is suggested that vaccination should be carried out annually, as in pre-transplant populations; though different dosing strategies have been tried, a firm recommendation regarding preferences over one dosing strategy versus another cannot be given [58].

### 3.2. Pneumococcal Vaccination

The AST IDCOP and Advisory Committee on Immunization Practices (ACIP) recommends pneumococcal vaccination for SOT recipients [4,37]. Recommendations include either the 20-valent pneumococcal conjugate vaccine (PCV20) or PCV21. Given that these conjugate vaccines provide broad coverage against pneumococcal serotypes, re-vaccination is not necessary. Alternatively, if the 15-valent pneumococcal conjugate vaccine (PCV15) is administered, then it is followed by the 23-valent polysaccharide pneumococcal vaccine (PPSV23) more than 8 weeks apart. If PPSV-23 was administered pre-transplant, a repeat dose is recommended at least 5 years after the initial dose.

### 3.3. Hepatitis B Vaccination

Post-transplant response to HBV is variable and often suboptimal due to immunosuppression. This underscores the importance of timely and appropriate pre-transplant HBV. High-dose vaccine series with Recombivax HB (40 µg) or Engerix-B (40 µg) may be preferred, though there are no consensus guidelines regarding this. Heplisav-B can also be given as a two-dose series. If anti-HBs titers are <10 mIU/mL 1–2 months after completing the vaccine series, revaccination can be considered [21,23]. SOT recipients receiving grafts from hepatitis core antibody (anti-HBc)-positive kidney donors are generally considered safe with a rate of seroconversion around 3.2% [59]; however, the risk is quite high in liver transplantation [60]. Antiviral prophylaxis varies across the transplant centers in this type of condition, and more consensus guidelines are required.

### 3.4. Human Papillomavirus

Female SOT recipients are at increased risk of developing Human Papillomavirus (HPV)-related diseases, including cervical intraepithelial neoplasia and other HPV-related cancers [40,41]. However, studies indicate that SOT recipients demonstrate suboptimal seroconversion for different HPV types. Factors include early post-transplant vaccination, lung transplantation, and higher tacrolimus levels associated with reduced immunogenicity [42].

Given these findings, current evidence suggests that standard HPV vaccination schedules may not provide optimal protection in this population. Future studies are needed to evaluate whether modified vaccination strategies, such as alternative timing relative to transplantation, additional booster doses, or enhanced immunogenic formulations, can improve immune responses and clinical protection in female SOT recipients.

### 3.5. Meningococcal Vaccination

Meningococcal vaccination is recommended for SOT recipients with specific risk factors for invasive meningococcal disease, such as asplenia or anticipated use of eculizumab. The AST IDCOP guidelines recommend that meningococcal vaccination should be completed at least 2 weeks before the initiation of eculizumab. However, despite vaccination, antibiotic prophylaxis is given due to the potential for suboptimal immune responses and incomplete strain coverage [4].

### 3.6. Tetanus–Diphtheria (Td) and Tetanus–Diphtheria–Acellular Pertussis (TdaP) Vaccines

The TdaP, DTaP, and Td vaccines can be safely administered to solid organ transplant recipients according to the same indications and schedules as the general population [4].

### 3.7. Respiratory Syncytial Virus

The recommendations for RSV vaccinations and prophylaxis in SOT recipients are based on recent advancements. In 2023, the Food and Drug Administration (FDA) approved two recombinant subunit RSV vaccines for the prevention of RSV-associated lower respiratory tract disease for SOT recipients aged ≥60 years. However, we recommend considering RSV vaccination for all solid organ transplant recipients, regardless of age, provided it is covered by insurance. Palivizumab is primarily recommended for high-risk pediatric SOT recipients under 24 months of age and is not typically recommended for adults [49].

### 3.8. Herpes Zoster

The recombinant zoster vaccine (RZV) is recommended for all SOT recipients given the increased risk for herpes zoster and its complications. The RZV can be administered at 3 to 6 months post-transplant [43,44].

### 3.9. Live Vaccines

**Figure 1:**
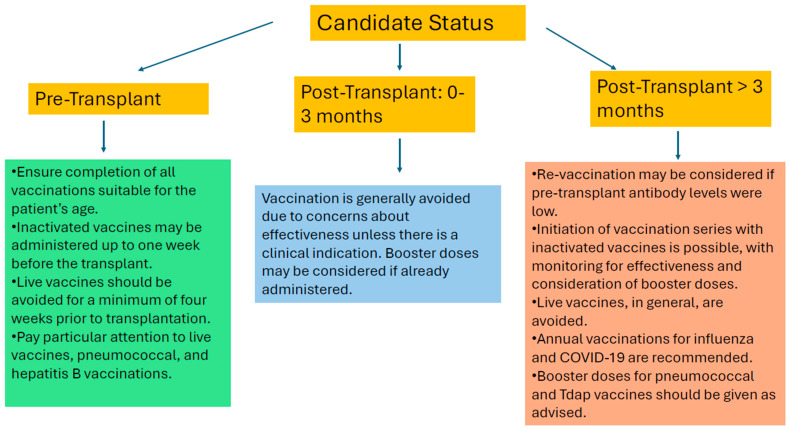
Recommendation for vaccination based on candidate status.

Live vaccines are generally contraindicated following SOT. However, emerging evidence suggests that certain live-attenuated vaccines, such as the varicella vaccine, may be safe for select post-transplant patients with low-level immunosuppression without recent allograft rejection [4,35,45,46]. Concerns remain regarding uncontrolled replication of the attenuated pathogens due to impaired immune response from immunosuppressive medications. This can result in severe infections, including life-threatening disseminated disease. A recent systematic review and meta-analysis encompassing pediatric and adult SOT recipients found that the varicella vaccine was generally well tolerated with a pooled seroconversion rate of 88.2% and negligible instances of vaccine-strain varicella disease [46]. Recipients were typically at least one year post-transplant, two months after a rejection episode, and on low-dose immunosuppressive therapy [46]. Similarly, another systematic review involving SOT recipients concluded that live vaccines, including the varicella vaccine, were generally safe when administered under low-level immunosuppression. The review found that most patients achieved satisfactory seroconversion rates with an uncommon occurrence of serious vaccine-related adverse events [45]. Consequently, vaccination with live-attenuated vaccines should be especially considered during outbreaks or travel to endemic regions, following a thorough assessment of the patient’s immune status and a careful weighing of the potential benefits and risks with the transplant team [4,35,45,46]. Table 3 reviews the seroconversion of vaccines post-transplantation. Figure 1 illustrates vaccination based on candidacy status.

## 4. COVID-19 Vaccination Pre- and Post-Transplantation, Including the Need for Yearly Vaccination

COVID-19 vaccination with BNT162b2 or mRNA-1273 has been associated with a lower risk of acquiring the infection as well as decreased risk of death and hospitalization in patients with end-stage renal disease [77]. In patients with chronic liver disease, COVID-19 vaccination has reduced the risk of developing the infection, though the immunogenicity of the vaccines appeared to be lower than in healthy individuals [78]. Cohort studies have shown that BNT162b2 mRNA and mRNA-1273 vaccines reduced the development of COVID-19 infection by 65% after the first dose and 79% after the second dose [79].

The immunosuppression in SOT patients increases the mortality and morbidity of COVID-19 infection in this population. COVID-19 vaccination is generally considered safe in SOT patients, but the response to the vaccine is limited given the immunosuppression [47]. Seroconversion rates to vaccinations against COVID-19 in SOT recipients were only 9% and 34% following the first and second dose in one systematic review [47]. The third dose improved response to about 66% in a subset, but not in all patients [47]. Therefore, a primary COVID-19 series in SOT patients either pre- or post-transplant is considered complete after three mRNA vaccines [48]. Given the limited efficacy, it is crucial that patients continue to follow safety measures post-transplant and seek timely care and management if they acquire the infection [47].

The clinical effectiveness of mRNA vaccines in SOT recipients is significantly limited when compared to the general population, but it may be improved by booster vaccine doses despite limited data [48]. However, the consensus is that SOT patients should receive age-appropriate booster vaccine doses regardless of how many doses they received in the past [48]. They should also be considered for yearly vaccination given the expected waning of immunity against the vaccine with time, new mutations, and their continued immunosuppression [48].

SARS-CoV-2 is constantly changing, and new variants and subvariants are expected to continue to emerge, while some variants may disappear (COVID-19 Data Tracker—CDC). The Omicron subvariant XBB of SARS-CoV-2 was first identified in the US in August 2022, and by January of 2023, it made up around 50% percent of cases across the US [80]. To evaluate vaccine efficacy against the emerging subvariants, a study reviewed data from the national Increasing Community Access to Testing, Treatment, and Response program (ICATT) and found that the relative vaccine efficacy of bivalent boosters among immunocompetent persons aged 18–49 years was around 52% against symptomatic BA.5 infection and 48% against symptomatic XBB/XBB.1.5 infection [80]. It was then concluded that staying current with recommended COVID-19 vaccination recommendations is advised as it continues to offer a good level of immunity [80]. The most recent COVID-19 vaccination schedule according to the Centers for Disease Control and Prevention for ages 12 years and older can be found on the website [81].

## 5. Special Situations

**Figure 2:**
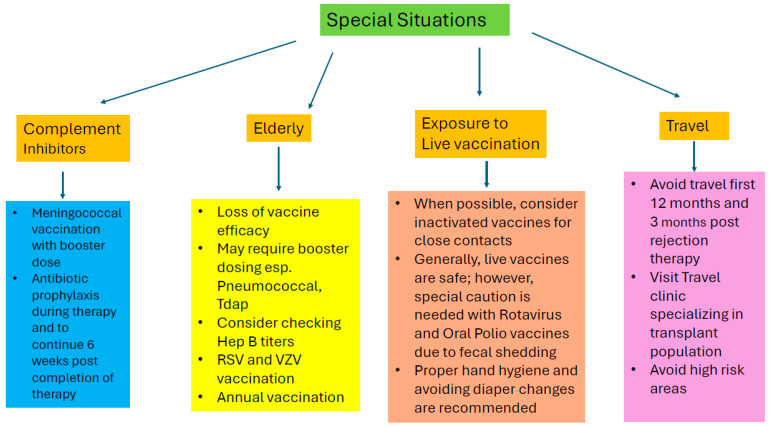
Vaccination in special situations.

Special vaccination considerations in solid organ transplant recipients include the use of complement inhibitors, the need for post-exposure prophylaxis, advanced age, and close contact with individuals or household pets receiving live vaccines; these scenarios are discussed in the following section (Figure 2).

a. Complement inhibitors

The inflammatory response generated by complement activation is an important cause of the tissue damage caused by antibody-mediated rejection in organ transplant recipients [82]. Downstream products of complement activation include biologically active protein complexes such as C5a and C5b-9 or the membrane attack complex (MAC). While MAC is responsible for the formation of pores through the cellular outer membranes, resulting in cell lysis and causing endothelial inflammation [83,84,85], C5a is needed for the upregulation of phagocytosis, an important mechanism in the destruction of encapsulated bacteria. The humanized monoclonal IgG antibody, eculizumab, binds to complement protein C5 and prevents its cleavage into C5a and C5b, thus inhibiting the formation of its terminal product, MAC. In addition to its use in aHUS, PNH, refractory myasthenia gravis, and neuromyelitis optica, eculizumab has also been used for perioperative desensitization and the prevention or treatment of AMR in kidney [86,87], heart [88], lung [89], and intestine [90] transplant recipients. The risk of life-threatening and fatal Neisseria meningitidis infection is increased up to 2000 times in those treated with complement inhibitors including eculizumab compared to healthy individuals, and has been included in the US FDA prescribing information for complement inhibitors as a black box warning. Similarly, recommendations are in place for the long-acting C5 inhibitor ravulizumab and the new C3 and C5 inhibitors, which may be used for different indications besides antibody mediated rejection. The CDC Advisory Committee on Immunization Practices recommends a complete vaccine series of MenACWY and MenB vaccinations and subsequent booster series throughout complement inhibitor therapy. It is recommended to complete or update meningococcal vaccination at least 2 weeks before the first dose of complement inhibitor [91] unless the risk of delaying the complement inhibitor therapy is outweighed by the risk of meningococcal disease. However, most often in our experience, physicians do not have the liberty to administer the vaccine 2 weeks before therapy starts, as eculizumab is most commonly initiated urgently for antibody-mediated rejection. In such cases, we recommend administering the vaccination just before the medication administration, if possible. We recommend administering the booster doses as per schedule to give antibiotic prophylaxis during the duration of therapy and continuing for at least 6 weeks post-therapy completion. Physicians must be enrolled in the REMS program to administer eculizumab or ravulizumab.

b. Elderly Population

The age distribution of solid organ transplant recipients varies by organ type. While >50% of end-stage renal disease patients are ≥65 years of age, they only constitute 3% of renal transplant recipients [92]. On the other hand, 19% [93] of heart transplant recipients, 23% of liver candidates [94], and 33% of those on the lung transplant wait list are ≥65 years of age [95]. The proportion of elderly people on solid organ transplant wait lists has nearly doubled from a decade prior, keeping pace with the aging world population. The short-term and medium-term outcomes of graft and overall survival are generally comparable in older and younger transplant recipients; however, cardiovascular disease and malignancies remain the main barriers to long-term survival. Aging also results in important changes in the immune system with altered innate immune response, exaggerated inflammation, and a decline in cell-mediated immunity [96,97]. The phenomenon described as immunosenescence results in a decrease in vaccine efficacy in elderly people [98]. There is both a reduced pool of initial vaccine-responsive cells and reduced survival of vaccine-specific memory cells. Several strategies to increase protective immunity in elderly individuals are recommended, including high-dose vaccines (influenza) [99], pneumococcal vaccination during hospitalization [100], and a multi-pronged strategy of using high-dose vaccines, multivalent vaccines, adjuvanted vaccines, and mRNA vaccines [101]. There are ongoing vaccine trials for inhibiting chronic inflammation or immunosenescence [101].

c. Post-exposure

Vaccinations given after exposure to a virus, known as post-exposure prophylaxis (PEP), aim to reduce disease severity or prevent disease entirely. This strategy is beneficial for several viral exposures, including measles, varicella zoster virus (VZV), smallpox, hepatitis A, influenza, and tetanus. For immunocompromised patients, if a live vaccine is the only option, immune globulins are the preferred PEP regimen [102]. Generally, PEP should be given as soon as possible following a high-risk exposure.

i. The MMR vaccine can serve as effective PEP for measles exposure, but it is not effective for mumps or rubella [103]. For non-immune individuals, the MMR vaccine should be administered within 72 h of exposure to prevent or mitigate measles infection [104]. For immunocompromised individuals, however, a single dose of immune globulin can be given from one day before rash onset to four days after rash resolution [102].
ii. Post-exposure VZV vaccination within five days of exposure is recommended for unvaccinated healthy individuals aged 12 months or older to prevent cutaneous lesions [102]. For immunocompromised patients at risk for severe complications, VZV immunoglobulin or antiviral therapy is recommended as PEP. This treatment should be initiated as soon as possible, up to 10 days post-exposure [102,103].
iii. The influenza vaccine can be used as PEP for unvaccinated individuals exposed from one day before symptom onset until one day after fever abatement, or potentially longer for immunocompromised patients [102]. High-risk patients with confirmed influenza may also be treated with antiviral agents [104].
iv. The tetanus vaccine is recommended as PEP for any individual with an incomplete vaccine history or if the most recent dose was administered more than 5–10 years prior, depending on wound cleanliness [102,105]. Tetanus PEP can also be administered several months after exposure due to the variable incubation period [102]. Tetanus is nearly entirely preventable with vaccination, and timely PEP significantly reduces disease severity [34].
v. The hepatitis A vaccine is recommended as PEP within 14 days of exposure [105]. Studies show that both the hepatitis A vaccine and immune globulins offer protection post-exposure, but the vaccine may provide longer-term immunity [106]. The hepatitis A vaccine is effective in preventing secondary infections and should be recommended for contacts of primary cases [107].

In the United States, updated COVID-19 vaccines are not typically used as post-exposure prophylaxis (PEP); instead, antivirals are generally administered. However, one study reported a significant reduction in mortality among patients vaccinated shortly after exposure compared with unvaccinated controls [108]. Emerging evidence suggests the need to further explore COVID-19 vaccines as potential PEP tools in specific cases.

Patients exposed to an infection and treated with PEP should be assessed at baseline and monitored while they remain at risk. Ensuring the completion of prophylactic regimens is crucial [102]. The current body of evidence highlights that there is still much to learn about post-exposure vaccination.

d. Contacts with pediatric population who undergo live vaccination

It is recommended that close contacts and family members of SOT patients be vaccinated, as these patients are at heightened risk of infection. The pediatric population may be exposed to live vaccines, as discussed below.

i. MMR vaccine: Viral shedding after MMR vaccination is not uncommon and may be detectable for up to 29 days in some individuals [109]. However, there is no evidence of human-to-human transmission of the measles vaccine virus, even among thousands of studies conducted worldwide [109]. Shedding from this vaccine is not considered a risk to SOT recipients [4].
ii. Varicella vaccine: Reactivation of the vaccine virus has been reported in rare cases, leading to conditions such as vaccine-associated rash, herpes zoster ophthalmicus, encephalitis, and meningitis [110]. Shedding occurs through vesicular fluid in cases where a rash develops post-vaccination [111]. Since 1995, only 11 immunocompetent vaccinated individuals have been documented to spread the virus to 13 unvaccinated contacts [112]. Covering lesions and practicing good hygiene minimizes transmission risk. Immunocompromised individuals should avoid contact with vaccinated individuals until the rash resolves, when possible.
iii. Rotavirus vaccine: The live-attenuated rotavirus vaccine can result in virus shedding in stool, particularly during the first week after vaccination [113,114,115]. Immunocompromised individuals should avoid contact, especially diaper changes, with vaccinated infants for up to four weeks, particularly for at least the first 14 days [113,116]. Rigorous hand hygiene, including the use of alcohol-based hand sanitizers, can help mitigate transmission risks [116,117].
iv. Live-attenuated influenza vaccine: The live-attenuated influenza vaccine (LAIV) is given annually to protect against the strains of influenza predicted to be most prevalent each year. Studies suggest that children tend to prefer the nasal spray over the intramuscular injection [118,119]. In September 2024, the FDA approved the FluMist nasal spray for both self-administration and caregiver administration, making it available for the 2025–2026 flu season. This approval could lead to increased use of FluMist across the US.
  Studies show that individuals, especially children, vaccinated with the live-attenuated influenza vaccine may shed the virus for up to 11 days post-vaccination [120,121,122]. However, while viral shedding is minimal and rarely leads to transmission [120,123], immunocompromised individuals should avoid close contact with those recently vaccinated with the live-attenuated flu vaccine. Close contacts of SOT patients should receive the inactivated influenza vaccine instead of LAIV. When this is not feasible, good hygiene practices, like frequent handwashing and covering the nose and mouth when coughing, can reduce transmission risks.
v. Oral polio vaccines: The oral polio vaccine is a live-attenuated vaccine and is not available in the US, but is available internationally in some countries. The oral polio vaccine was replaced by the inactivated polio vaccine in 2000 [124]. The oral polio vaccine has the potential for viral replication and shedding; therefore, SOT patients should avoid contact with vaccine recipients’ stool, unhealed vaccination sites, or bandages during the first four weeks post-vaccination [125,126].
vi. Vaccination strategies for close contacts: To protect SOT recipients, the vaccination of close contacts—family members and healthcare workers—is critical. Whenever possible, inactivated vaccines should be used to minimize exposure risks [4]. When unavoidable, strict adherence to proper hygiene practices and the avoidance of close contact during periods of viral shedding are important to reduce the risk of transmission to immunocompromised patients.

e. SOT recipients with pets who receive live vaccination

The presence of pets in the home provides significant emotional and mental health benefits, but it also requires consideration of potential health risks, especially for immunocompromised individuals [127]. Some animals, such as reptiles, poultry, and rodents, are considered higher risk compared to dogs and cats [128]. Pets that receive live vaccines can occasionally shed the vaccine strain in their saliva, urine, or feces, posing a possible risk of zoonotic transmission. While this risk is minimal, immunocompromised individuals may be more susceptible [129].

Human disease due to pet-associated zoonoses is generally considered rare; the true incidence is not well known due to sporadic cases that are often unreported [130]. It is generally recommended that immunocompromised individuals avoid any contact with vaccinated pets and their excretions for up to six weeks after vaccination [129].

Just as with human vaccines, immunocompromised patients should opt for non-live vaccines for their pets [129]. Additionally, they should minimize direct contact with pet bodily fluids, avoid bites or scratches, and maintain proper hand hygiene to reduce risks [130,131]. However, studies on the transmission of zoonotic agents through live vaccines in pets are limited [127], and research is needed to assess the full extent of potential risks. A survey of physicians and veterinarians revealed that while veterinarians should play a key role in advising pet owners about zoonotic risks, patients do not always view veterinarians as the primary source of this information [127]. Further studies are warranted to clarify the potential risk of contracting zoonotic disease transmission from live vaccines in pets.

## 6. Vaccine Response Concerns Pre- and Post-Transplant

The use of immunosuppressive medications post-transplant predisposes recipients to infections that may otherwise be preventable. However, it is important to note that an immunosuppressed state also exists pre-transplant due to end-stage organ disease, which increases their susceptibility to various infections [132]. This immune system dysfunction is a result of dysregulated innate and adaptive immune responses with reduced expression of Toll-like receptors, reduced B cell and T cell proliferation, impaired leukocyte and endothelial function, and the presence of an inflammatory state. Pre-existing comorbid conditions such as diabetes mellitus and severe protein calorie malnutrition, or the concurrent use of immunomodulatory medications for the management of end-stage organ disease, also increase susceptibility to infections and may affect the immunogenicity of vaccinations. Therefore, it is important to consider vaccinations for preventable diseases in both organ transplant candidates and recipients, but vaccinations before and after solid organ transplantation bring up several important questions, which are discussed here.

a. What is the ideal timing for vaccinations?

There is a general consensus that the best time to vaccinate is pre-transplant, and a systematic approach to making vaccinations part of the transplant candidate evaluation process is recommended [133]. This is particularly important for immunization with live attenuated vaccines (MMR, Yellow Fever, varicella, herpes zoster, oral typhoid, oral polio, rotavirus) as they are often avoided in the post-transplant phase. However, the science on live vaccination post-transplantation is evolving, as discussed above. The AST clinical practice guidelines recommend that live virus vaccination precede organ transplantation by a minimum of 4 weeks [4]. The reported vaccine-related infection rate is 4.7% and infections are generally mild to moderate without any mortality [45]. Serious risk–benefit assessment is needed before the administration of live attenuated vaccines in organ transplant recipients. The absence of primary vaccination and the usually higher immunogenicity of live attenuated vaccines in pediatric solid organ transplant recipients suggest greater benefits than harms in children, with mounting evidence regarding the safety of MMR and varicella vaccines post-transplant. Other live vaccines like polio, oral typhoid, and inhaled influenza vaccine remain contraindicated post-transplant [134].

Inactivated vaccines are safe during the post-transplant phase, but both primary and secondary vaccine series should be delayed by 3–6 months after transplant, with a possible need to repeat the dose after 1 year to achieve maximum immunogenicity [4]. A notable exception to this is the influenza vaccine, which may be given as early as 1 month post-transplant, especially in lung transplant recipients, but may need to be repeated in 3 months to achieve the best effect.

b. Is the assessment of vaccine response needed?

Assessing the serologic response to vaccination should be considered a standard part of vaccination practices before transplantation, but data on the post-transplant monitoring of serologic response remain a subject of debate. Response rates to vaccines in SOT recipients are generally 10–16% lower than in healthy controls [64]. Several factors determine the immunogenicity to vaccines including the type of vaccine (tetanus and diphtheria vaccines elicit a response comparable to healthy controls, while hepatitis A and B vaccines have significantly reduced response in SOT recipients), type and dose of immunosuppressive agent (SOT recipients had better response to calcineurin inhibitors and azathioprine than sirolimus and mycophenolate), level of net immunodeficiency, and host factors such as age of the recipients. The duration of the protective response is less understood, as there is a paucity of studies with >12 months of response data. The initial vaccine response is a strong predictor of the duration of protective effects. In a study of renal transplant recipients that evaluated the response to hepatitis B vaccination, about 25% of recipients had lost protective anti-HBsAb by 12 months, and the majority (93%) of those who maintained protective titers had a robust initial response (≥100 IU/L) at baseline after vaccination [135]. However, data is currently lacking to support revaccination against most diseases after transplant, but the benefits of revaccination may outweigh any risks involved in most transplant recipients. Therefore, revaccination after 12 months of the initial dose should be considered if the infection risk remains.

The ideal timing of assessing vaccine response is unknown, as assessing the response too early may result in low titers, while waiting several months after vaccination may also be unhelpful, since the natural decline in antibody titers will render it difficult to assess whether the recipient had a suboptimal response to vaccination. The serologic titers of vaccine-induced antibodies may be assessed a minimum of 4 weeks after vaccination [4]. An additional consideration should be made if the organ recipient has received replacement intravenous immune globulins, either as part of the treatment for a rejection episode or for treatment or prevention of certain infections. The reliability of serologic assessment in the presence of other immunobiologics is questionable, as antibodies may be of either donor or recipient origin.

Not all vaccines require the monitoring of serologic response. The vaccines that are recommended for evaluation include the inactivated vaccines hepatitis A, hepatitis B, H influenza type B, and rabies; and the live-attenuated vaccines varicella (Varivax) and MMR [4].

c. Does vaccination pre- or post-transplant increase the risk for rejection?

As with natural infections, the immune response to vaccine antigens can theoretically produce cross-reactivity with alloantigens. Additionally, the adjuvants used in vaccines may also stimulate the immune system non-specifically and possibly result in allosensitization. The safety of vaccinations after a solid organ transplant has been a topic of intense debate, and several small studies and meta-analyses have addressed this. A large meta-analysis on this topic by Mulley et al. included 15,645 vaccinated transplant recipients and 42,924 control patients. The overall incidence of rejection episodes was 2.1%, with no increase in rejection risk reported with vaccination compared with non-vaccinated individuals [136]. An increase in annual de novo donor-specific antibody (DSA) (7–22% in vaccinated vs. 2.5–5% in controls) was reported for kidney, liver, and heart transplant recipients, but not lung transplant recipients (up to 17% in controls). Despite this slight increase in de novo DSA, no increase in allograft loss incidence was noted in vaccinated individuals for up to 12 months following vaccination.

Although there are no clear predictors of DSA response to vaccinations, individual vaccines have been evaluated as a potential cause. A cohort of solid organ transplant recipients who were vaccinated with pandemic-adjuvanted influenza vaccines had an increase in the development of de novo anti-HLA antibodies, more prominent with the second vaccination dose, but only 1% of these were donor-specific antibodies and no graft rejection was noted in this group [137]. Similarly, no graft-directed immune activation has been found following SARS-CoV-2 vaccination [138]. There is some evidence that females may experience greater non-specific HLA antibody induction after vaccination compared to males [139]. Overall, current evidence suggests the safety of vaccinations with no significant allograft-directed de novo antibody development in organ transplant recipients.

## 7. Travel Vaccination

Immunocompromised hosts are not excluded from the increasing rates of global and adventurous traveling worldwide [140]. A review of 1130 SOT recipients showed that 27% traveled outside the US and Canada, with 49% traveling to destinations that are considered high risk [141]. There are low rates of pre-travel vaccination in this population, with low rates of following mosquito avoidance measures [140]. Engaging in high-risk activities while traveling has been reported among immunocompromised patients [140].

Transplant recipients are at increased risk for vaccine-preventable infectious diseases [4,140]. Patients should be advised to avoid traveling, especially to high-risk destinations, in the first 12 months post-transplant [140]. We also recommend avoiding travel immediately following rejection therapy, though there is no specific recommendation, and waiting time may depend on the type of rejection and therapy administered. It also depends on the graft status, net degree of immunosuppression at the time of travel, and knowledge of endemic infections.

Advice about traveling post-transplant and its risks should be part of routine pre-transplant evaluation and counseling [142]. SOT recipients who wish to travel after transplant should be highly advised to consult a travel care specialist before traveling, especially before visiting a high-risk destination [142]. Patients are encouraged to visit the travel clinic several months before their planned travel as vaccinations take several months to complete and to ensure maximum effectiveness before traveling [142]. Evaluating serological response after vaccinations to determine whether a repeat dose is needed has not been studied in the SOT population and is generally not recommended [142]. Table 4 provides guidance for travel-related vaccinations.

## 8. Current Vaccine Trials

As of December 2024, there are over fifty active vaccine-related clinical trials listed on ClinicalTrials.gov covering a range of topics, including COVID-19, shingles, RSV, rotavirus, influenza, and immunization for special populations such as immunocompromised individuals. However, only a limited number of these trials focus specifically on SOT recipients (Table 5). These trials are critical in addressing key questions about vaccine safety and immunogenicity in SOT recipients. Due to immunosuppression, SOT patients often have suboptimal vaccine responses and increased risk of complications. These current trials aim to evaluate factors such as optimal vaccine dosing, timing of administration, and the use of different formulations to improve immune responses.

Additionally, these studies will help establish the safety and efficacy of newer vaccines and provide evidence-based guidance on vaccine scheduling in relation to transplantation. For instance, determining ideal timing for administration—whether pre-transplant for immunogenicity or post-transplant to minimize graft rejection risk—is a key area of focus.

## 9. Conclusions

Vaccination remains a critical component of comprehensive care in SOT recipients, balancing infection prevention with immunological safety. Pre-transplant immunization provides an essential opportunity to establish protective immunity while minimizing risks associated with live-attenuated vaccines. Post-transplantation, vaccination strategies must be individualized, accounting for immunosuppressive regimens, time since transplant, and serological response.

Transplant teams must work closely with infectious disease specialists to ensure that SOT recipients are appropriately protected without compromising graft function or patient safety.

A proactive, evidence-informed, and patient-centered approach to vaccination can reduce morbidity and mortality in this vulnerable population, reinforcing the pivotal role of preventive medicine in long-term transplant success.

## Figures and Tables

**Table 1:** Pre-transplant pneumococcal vaccine schedule in solid organ transplantation for unvaccinated individuals *.

Prior Pneumococcal Vaccination Status	Age/Risk Group	Recommended Vaccination Strategy
None or PCV7 only	Adults aged ≥50 years	PCV21 or PCV20 (single dose), or PCV15 followed by PPSV23 ≥ 1 year later ^a^
	Adults 19–49 years with immunocompromised conditions ^b^, CSF leak, or cochlear implant	PCV21 or PCV20 (single dose), or PCV15 followed by PPSV23 ≥ 8 weeks later
	Adults 19–49 years with chronic medical conditions ^c^	PCV21 or PCV20 (single dose), or PCV15 followed by PPSV23 ≥ 1 year later
PPSV23 only	Adults aged ≥50 years	PCV21, PCV20, or PCV15 ≥ 1 year after PPSV23
	Adults 19–49 years with immunocompromised conditions ^b^, CSF leak, or cochlear implant	PCV21, PCV20, or PCV15 ≥ 1 year after PPSV23
	Adults 19–49 years with chronic medical conditions ^c^	PCV21, PCV20, or PCV15 ≥ 1 year after PPSV23
PCV13 only	All adults aged ≥19 years with an indication for pneumococcal vaccination	PCV21 or PCV20 ≥ 1 year after PCV13
PCV13 + PPSV23 (PPSV23 given at age < 65 years)	Adults aged ≥50 years	PCV21 or PCV20 ≥ 5 years after last pneumococcal vaccine
	Adults 19–49 years with immunocompromised conditions ^b^	PCV21 or PCV20 ≥ 5 years after last pneumococcal vaccine (series complete)
PCV13 + PPSV23 (PPSV23 given at age ≥ 65 years)	Adults aged ≥65 years	Shared clinical decision-making: PCV21 or PCV20 ≥ 5 years after last pneumococcal vaccine
PCV13 + 2 doses of PPSV23	Adults 19–49 years with immunocompromised conditions ^b^	Reassess at age 50 years or PCV21 or PCV20 ≥ 5 years after last dose (series complete)
PCV13 + PPSV23	Adults 19–49 years with chronic medical conditions ^c^	Reassess pneumococcal vaccination status at age 50 years

* Adapted from Advisory Committee on Immunization Practices, 2025 [11]. ^a^: For adults who have received PCV15 but have not completed their recommended pneumococcal vaccine series with PPSV23, 1 dose of PCV21 or PCV20 may be used if PPSV23 is not available. ^b^: End-organ dysfunction, immunosuppressed status, cancer congenital or acquired asplenia, or sickle cell disease or other hemoglobinopathies. ^c^: Alcoholism, major organ dysfunction, asthma, cigarette smoking, or diabetes mellitus.

**Table 2:** Post-transplant vaccination. The table summarizes the key post-transplant vaccines and their types, timing, and rationale for adult solid organ transplant recipients.

Vaccine	Type	Timing Post-Transplant	Comment
Influenza [4]	Inactivated	1 month onwards, annually	High morbidity and mortality reduction from influenza infection
Pneumococcal [4,37]	Conjugate (PCV-20 or PCV-21)	3 months onwards	Increased serotype coverage
Hepatitis B [21,23]	Inactivated	3 months onwards	Post-transplant response to HBV is variable; booster if follow up anti-HBs titers are <10 mIU/mL
Hepatitis A [38,39]	Inactivated	3 months onwards	Post-transplant response to HAV vaccination is variable
Human Papillomavirus [40,41,42]	Inactivated	3 months onwards	Prevention of HPV-related cancers
Meningococcal [4]	Conjugate	6–12 months onwards	Two weeks prior to anticipated use of eculizumab, asplenia with continued antibiotic prophylaxis during and for at least 6 weeks post completion of therapy
Tdap, DTaP, and Td [4]	Inactivated	3 months onwards	Same indications and schedules as the general population
Herpes Zoster [43,44]	Adjuvanted recombinant zoster vaccine (RZVor Shingrix)	3–6 months onwards	Increased risk for herpes zoster and its complications. Ideally, vaccination should be completed pre-transplant. Avoid during acute infection
Varicella [45,46]	Live-attenuated	Case-by-case	Emerging safety data for select patients under low-level immunosuppression
Measles, Mumps, Rubella [45]	Live-attenuated	Case-by-case	Emerging safety data for select patients under low-level immunosuppression
COVID-19 [47,48]	Inactivated (m-RNA)	3 months onwards	See Section 4
RSV [49,50]	Inactivated (recombinant, subunit RSV vaccine)	3–6 months onwards	FDA approval for recombinant subunit RSV vaccines for the prevention of RSV-associated lower respiratory tract disease for SOT recipients aged ≥60 years. Palivizumab is not recommended for adults

**Table 3:** Immunogenicity/serologic response ranges in solid organ transplant recipients.

Vaccine	Efficiency (Seroconversion)	Comment
Influenza [61,62,63]	30–62%	Seroconversion rates vary substantially, though improved over the years
Pneumococcal (PCV20/21)	Direct efficacy data limited	Immunogenicity observed in PCV13 studies and general efficacy data in adult population supports use
Hepatitis A [64,65]	0–67% after the first dose; 0–97% after the second dose	Significant variability depending on the type of SOT and immunosuppression regimen. Overall immune response is lower compared to healthy populations
Hepatitis B [66,67]	36% to 76.5%	Variable efficacy. Higher rates observed when vaccination is completed pre-transplantation
Human Papillomavirus	Not well defined	HPV efficacy rates in solid organ transplant recipients are less clear and typically lower
Meningococcal [68]	Not well defined, 40% in kidney and liver transplant recipients	Responses are generally low and suggest that additional measures such as booster doses or alternative vaccination schedules may be necessary
Tdap, DTaP, and Td [69]	88.5–100%	Antibody levels my decrease after a year post-transplantation, necessitating booster doses, particularly for diphtheria
Herpes Zoster [70,71,72,73]	55–67%	For recipients of recombinant zoster vaccine (Shingrix) seroconversion rates are lower than efficacy observed in healthy individuals (>90%). Ideally, vaccination should be completed pre-transplant
Varicella [33]	33–50%	Lower seroconversion rates for varicella vaccination post-SOT
Measles, Mumps, Rubella [33]	50–89%	Recent reports indicate lower response rates for the MMR vaccination–seroconversion for measles ~66.7%. Emerging data for case-by-case consideration in select patients
COVID-19 [74,75] (mRNA)	18–67%	Seroconversion rates among SOT recipients can be significantly lower or delayed compared to healthy populations, emphasizing the need for booster doses
RSV [76]	67% seroconversion rate in lung transplant recipients, overall data on SOT recipients are still limited	Arexvy^®^ (RSVPreF3) recombinant subunit vaccine in lung transplant recipients demonstrated significant immunogenicity with sustained antibody responses. Ongoing studies necessary to assess broader applicability across different types of SOT recipients

**Table 4:** Travel-related vaccine recommendations for solid organ transplant recipients * [142].

Travel Related Vaccine	Recommendations
Hepatitis A	Recommended for all travelers based on risk assessment
Meningococcal conjugate	Recommended if not already administered pre-transplant based on risk assessment
Meningococcal serogroup B	Recommended if not already administered pre-transplant based on risk assessment
Inactivated polio (IPV)	All travelers with h/o complete a primary series of polio vaccine with one additional lifetime dose of IPV given to adults above the age of 18 years
Rabies	Recommended if likely to have significant exposure to animals including hunting abroad. Consider post-exposure prophylaxis
Japanese encephalitis	Recommended when indicated based on risk assessment
Cholera vaccine	Recommended when indicated based on risk assessment. Contraindicated in the US for immunocompromised individuals as live vaccine is the only available vaccine (Vaxchora)
Typhim Vi	Recommended when indicated based on risk assessment
S typhi Ty21a	Contraindicated as it is a live vaccine
Oral polio (OPV)	Contraindicated as it is a live vaccine
Bacille Calmette–Guerin	Contraindicated as it is a live vaccine
Yellow Fever	Contraindicated as it is a live vaccine
Dengue Fever	Recommend to use mosquito repellants, full body clothing, and bed nets

* Adapted from American Society of Transplantation Infectious Diseases Community of Practice [142].

**Table 5:** Current vaccine trials in solid organ transplantation.

Trial Name	NCT Number	Status	Population	Vaccine/Intervention	Study Design	Primary Outcomes
Recombinant Zoster Vaccine in Young Adult Solid Organ Transplant Recipients	NCT06162494	Not Yet Recruiting	Young adult solid organ transplant (SOT) recipients	Recombinant zoster vaccine (RZV)	Open-label	Safety and immunogenicity of RZV; antibody and cellular immunity at baseline, 1–2, 6, and 12–15 months
Immunogenicity of HPV Vaccine in Transplant Recipients	NCT05557370	Recruiting	Post-SOT recipients	Gardasil 9 (HPV vaccine)	Prospective, open-label cohort	Change in geometric mean titers (GMT) at 7, 12, and 24 months post-vaccination
COVID-19 Booster and IIV Schedule in Immunocompromised Hosts (CO2I2)	NCT06599658	Recruiting	Immunocompromised individuals	COVID-19 booster + influenza vaccine	Phase II, randomized controlled trial	Immunogenicity based on co-administration vs. sequential timing and different intervals
Induction of Immunity Against Measles in Pediatric Liver Transplant Recipients (MMRinOLT)	NCT01770119	Recruiting	Pediatric liver transplant recipients	MMR vaccine	Observational study	Measles-specific antibodies and efficacy of additional MMR doses
High vs. Standard Dose Influenza Vaccine in Adult SOT Recipients	NCT04613206	Active, Not Recruiting	Adult SOT recipients, 1–11 months post-transplant	High-dose vs. standard-dose influenza vaccine	Multicenter, phase II randomized trial	Prolonged immunogenicity and hemagglutination inhibition (HAI) titers
Evaluating Immune Response to COVID-19 Vaccines in Patients With Cancer, Transplant or Cellular Therapy Recipients	NCT05164016	Active, Not Recruiting	Cancer patients, transplant or cellular therapy recipients	COVID-19 vaccines	Observational study	Antibody and T-cell response; COVID-19 infection severity and immune response durability
Additional Recombinant COVID-19 Humoral and Cell-Mediated Immunogenicity in Immunosuppressed Populations	NCT06027229	Active, Not Recruiting	Immunosuppressed patients (IBD, SOT recipients)	COVID-19 recombinant booster vaccines	Observational study	Sustained humoral and cell-mediated responses; 1-month and 6-month antibody levels

## Data Availability

No new data were created or analyzed in this study. Data sharing is not applicable to this article.

## Abbreviations

ACIP	Advisory Committee on Immunization Practices
AST	American Society of Transplantation
AST IDCOP	American Society of Transplantation’s Infectious Diseases Community of Practice
CDC	Centers for Disease Control
CKD	chronic kidney disease
DSA	donor-specific antibody (DSA)
ESRD	end-stage renal disease
FDA	Food and Drug Administration
HBV	hepatitis B vaccination
HPV	Human Papillomavirus
LAIV	live-attenuated influenza vaccine
MMR	measles, mumps, and rubella
PEP	post-exposure prophylaxis
RSV	respiratory syncytial virus
RZV	recombinant zoster vaccine
SOT	solid organ transplant
Td	tetanus–diphtheria
TdaP	tetanus–diphtheria–acellular pertussis
VZV	varicella zoster virus
